# Fault Diagnosis for Power Transformers through Semi-Supervised Transfer Learning

**DOI:** 10.3390/s22124470

**Published:** 2022-06-13

**Authors:** Weiyun Mao, Bengang Wei, Xiangyi Xu, Lu Chen, Tianyi Wu, Zhengrui Peng, Chen Ren

**Affiliations:** 1 State Grid Shanghai Electric Power Research Institute, Shanghai 200437, China; maoweiyun1991@163.com (W.M.); xuxiangyi_1986@126.com (X.X.); chenlu8766@163.com (L.C.); wtyfenghua@163.com (T.W.); skanson@163.com (Z.P.); 18901658588@163.com (C.R.); 2 State Grid Shanghai Municipal Electric Power Company, Shanghai 200122, China

**Keywords:** semi-supervised transfer learning, fault type diagnosis of power transformers, three-phase grounding current of the iron core, deep neural network

## Abstract

The fault diagnosis of power transformers is a challenging problem. The massive multisource fault is heterogeneous, the type of fault is undetermined sometimes, and one device has only met a few kinds of faults in the past. We propose a fault diagnosis method based on deep neural networks and a semi-supervised transfer learning framework called adaptive reinforcement (AR) to solve the above limitations. The innovation of this framework consists of its enhancement of the consistency regularization algorithm. The experiments were conducted on real-world 110 kV power transformers’ three-phase fault grounding currents of the iron cores from various devices with four types of faults: Phases A, B, C and ABC to ground. We trained the model on the source domain and then transferred the model to the target domain, which included the unbalanced and undefined fault datasets. The results show that our proposed model reaches over 95% accuracy in classifying the type of fault and outperforms other popular networks. Our AR framework fits target devices’ fault data with fewer dozen epochs than other novel semi-supervised techniques. Combining the deep neural network and the AR framework helps diagnose the power transformers, which lack diagnosis knowledge, with much less training time and reliable accuracy.

## 1. Introduction

This section illustrates the research’s motivation, target and framework, followed by a relevant literature review of the recent research about the fault diagnosis of power transformers. After that, we state the research gap, our contributions and this paper’s organisation.

### 1.1. Motivation, Target and Framework

Power transformers are important equipment in electrical power systems, and transformer failures will negatively impact whole systems. An early-stage diagnosis of transformer fault type can save the high cost of repairing and downtime. The three-phase grounding currents of the iron core generated during the turning on of transformers are important indexes to assess the states of power transformers. The value of the normal grounding current of the iron core is quite small, while the abnormal grounding current value will increase substantially. The warning threshold for the grounding current of the iron core is 100 mA for the 110 kV power transformers and the faults are caused by Phases A, B, C and ABC to ground fault. This research aims to construct a reliable model that can analyse the fault type after 10 min from fault occurrence based on the abnormal three-phase grounding current data of the iron core, and the model can be quickly transferred to different devices with quick retraining to obtain good performance.

Benefiting from the evolution of machine learning, more and more supervised or unsupervised models have been adopted to pinpoint the linear or nonlinear relationships between electrical features and fault types [1]. This paper proposes a new fault diagnosis framework based on convolutional neural networks (CNN) [2,3,4], an attention mechanism [5] and bidirectional long short-term memory networks (BiLSTM) [6,7].

Besides that, we compose a new framework of semi-supervised transfer learning called adaptive reinforcement (AR). This technique aims to solve the problem of unbalanced and undefined fault type data in the target power transformer, which negatively affects the transferring process. The first step of AR is pretraining the source model’s classifier and then giving pseudolabels to the unlabelled data by the trained model. After that, we supplement the unbalanced fault data from the source domain based on the probability distribution. To overcome the problem of overfitting the complemented data, we replay the training experience of the main type of fault. Finally, we fine-tune the whole network’s weights with few epochs to fit the target device.

**Figure 1:**
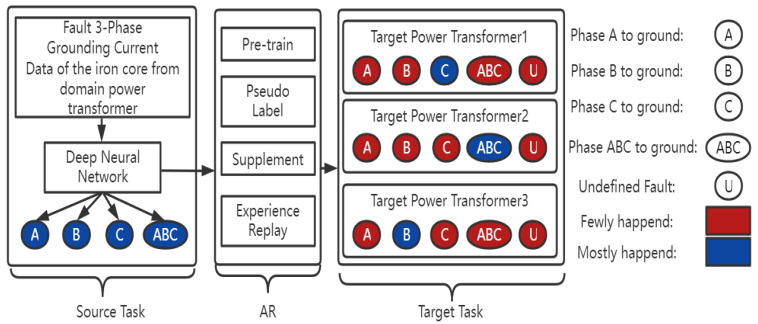
Methodology of the whole framework.

The comprehensive framework of the methodology is shown in Figure 1. The source domain’s data are the three-phase fault grounding current data of the iron core after the fault happens from one 110 kV main power transformer with the corresponding four types of faults: Faults A, B, C and ABC to ground. In addition, the fault classes are balanced. We train the proposed deep neural network to classify the fault type and then transfer the model to the target domain in the framework of AR. There are three 110 kV target power transformers for the target task, and each of them contains undefined fault data. Moreover, the fault types in the target power transformer are unbalanced. The blue color indicates the fault type which mostly happened in that device, the red color indicates the fault type which rarely happened and U indicates the undefined fault type.

### 1.2. Literature Review

Research on fault diagnosis and the detection of power transformers focuses on different aspects and methodologies, separately. The frequency response analysis (FRA) technique is one main method to detect transformer tap changer faults. The experiment in [8] was conducted on a three-phase 50 Hz, 11/0.415 kV, 500 kVA distribution transformer with a de-energized tap changer and simulated the tap changer fault to use FRA measurements to identify various contact faults such as coking and pitting. The work in [9] was conducted on a three-phase 11/0.433 kV, 500 kVA distribution transformer and calculated the correlation coefficient of the deviation from the fingerprint signature to identify the fault type. The work in [10] used the correlation between physical circuit parameters and various faults and quantified the impact of each fault with respect to the healthy signatures at different frequency regions. The work in [11] proposed a deep-learning-based framework named SigdetNet, which takes the power spectrum as the network’s input to localize the spectral locations of the signals. The research in [12] proposed an intrinsic time-scale decomposition (ITD)-based method for power transformer fault diagnosis based on dissolved gas analysis (DGA) parameters and used an XGBoost classifier to classify the optimal PRC feature set. The work in [13] proposed an artificial neural network (ANN) to establish the power transformer fault classification based on DGA. The work in [14] proposed a Hilbert–Huang transform (HHT)-based algorithm to effectively detect malfunctioning by revealing the instantaneous amplitude and frequency of nonlinear and nonstationary signals. The work in [15] employed the algorithm using principal component analysis (PCA) to easily find the feature of the fast Fourier transform (FFT) signal and utilize $Hotelling’sT^{2}$ as an index for fault detection. The work in [16] adopts the nonstationary signal processing techniques: wavelet transform (WT) to the application of industrial equipment and rotating machinery fault diagnosis (RMFD).

A well-trained model being adapted to similar tasks has become a popular technique with the advantages of saving time and having better model performance in the engineering area. Paper [17] split the transfer learning work fault diagnosis into four categories: feature, GAN (generative adversarial network), example and parameters. The GAN-based transfer method generates fake samples, which is not suitable for our task, so we reviewed the other three types’ work here. Transfer learning for time series classification [18] adopted a fully convolutional neural network model to pretrain UCR archive datasets. It then transferred the learned features (the networks’ weights) to a second network to be fine-tuned on a target dataset. In this paper, fine-tuning is still the main method while transferring the model. Domain adaption adjusts the weights of the pretrained model by the decision boundary between the source and target domains. Online and Offline Domain Adaptation for Reducing BCI Calibration Effort [19] proposes an algorithm that originates from the work in [20] that adapts the weights offline and online for unlabelled target data tasks. Adaptive Consistency Regularization for Semi-Supervised Transfer Learning [21] proposes adaptive consistency regularization. It adapts the model weights based on the consistency between the source model and data with the target model and data. Transfer Learning Based Fault Diagnosis with Missing Data Due to Multi-Rate Sampling [22] transfers the pretrained model from the missing sensors’ domain to complete data by completing the corresponding missing weights with random initial values. The work in [23] imposed a Lautum-information-based regularization that relates the network weights to the target data. The work in [24] offered a free-distribution-, kernel- and graph-Laplacian-based approach which optimizes empirical risk in the appropriate reproducing kernel Hilbert space. The work in [25] proposed an expectation-maximization algorithm to solve the transferred model iteratively.

### 1.3. Research Gap

The literature review explored several novel works on fault diagnosis and transfer learning. FPA-based methods mostly focus on transformer tap changer faults by calculating the correlation coefficients. Deep-learning-based and machine-learning-based frameworks mostly utilize dissolved gas analysis (DGA) to establish fault classification. Transforming methods, including the Hilbert transform, fast Fourier transform and wavelet transform, study the information contained in the frequency to predict the occurrence of the fault. For transfer learning, fine-tuning is still the most popular method. For semi-supervised transfer learning, the novel proposed techniques are mostly based on the ideas of target domain adaption and consistency regularization.

This paper proposes a deep neural network to figure out the fault types of power transformers. The architecture is formed by the FCNN, attention mechanism and BiLSTM. For the semi-supervised transfer learning task, AR enhances the domain adaption step by mixing the dynamic time wrapping algorithm. In addition, the experience replay step reinforces the experience of training the major fault.

### 1.4. Contribution

Our main contributions can be summarized below:

1. To the best of our knowledge, we are the first to combine the semi-supervised transfer learning method with the experience replay idea and utilize this method to solve electrical system problems.
2. We combine fully convolutional neural networks (FCNN) with an attention mechanism followed by bidirectional long short-term memory (BiLSTM) networks as the diagnosis model’s architecture to classify the fault type accurately.
3. Considering the unbalanced and undefined fault data in the target task, we propose an enhancement on the pseudolabel method based on cross-entropy and dynamic time wrapping algorithms.

### 1.5. Organization

The rest of the paper is organized as follows: Section 2 introduces the data and experiment’s setup, and Section 3 details the architecture of the deep neural network. Section 4 presents the framework of semi-supervised transfer learning. The evaluations of the experiments are carried out in Section 5. Section 6 discusses the results. The conclusion is drawn in Section 7.

## 2. Materials and Methods

This section specifies the characteristics of the datasets, exploration methods and preprocessing steps as the setup for the experiments.

### 2.1. Dataset and Exploration

The datasets we employed for this research were the 3-phase fault grounding currents of the iron core data created by the main 110 kV power transformers owned by State Grid Shanghai Municipal Electric Power Company. We recorded the fault current after it reached the warning threshold, which was 100 mA. The source domain samples were the fault grounding current data of the iron core by one 110 kV main power transformer produced last year with four types of faults: Phase A to ground, Phase B to ground, Phase C to ground and Phase ABC to ground. The target domain samples were three different 110 kV power transformers’ fault data which contained labelled and unlabelled fault types. The value of the normal grounding current of the iron core was below 10 mA, and the abnormal current value increased substantially to about 150 mA. After the fault happened, as the abnormal current value appeared, we waited until the fault data had enough length and then input it into the model to classify the fault type.

#### 2.1.1. Source Domain

The source data were collected from one main power transformer. Three detection devices monitored Phases A, B and C’s grounding currents of the iron cores individually. The detection devices recorded the corresponding grounding current data of the iron core simultaneously every twenty seconds. The maintenance worker found out the fault’s reason and recorded it each time.

We selected the fault data $D_{s}=\left\{X_{1,2...n}\right\}$, where the number of time-steps n=30 with their corresponding fault types as the source dataset samples. We found that the whole-time length from the beginning of the fault happening until fixing was always more than one hour. In order to have enough training data for a deep neural network, we chose the abnormal phase grounding current data of the iron core from the beginning of faults every 10 min.

**Figure 2:**
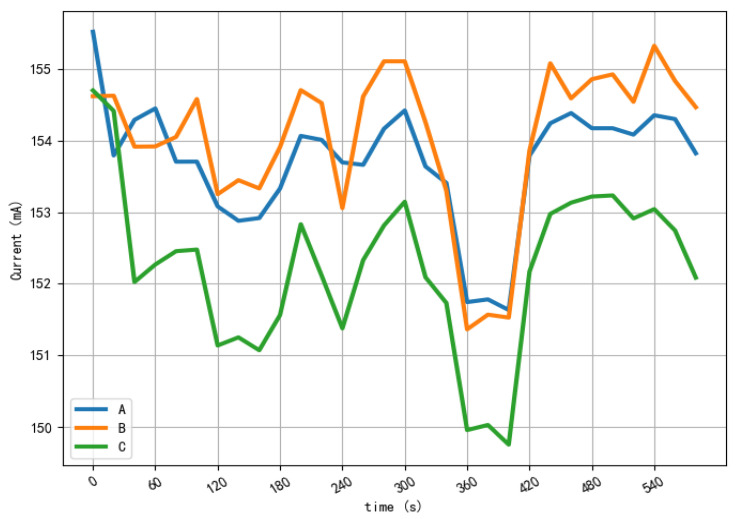
One sample of the 3-phase grounding current of iron core data with the fault of Phase A to ground.

Figure 2 shows an example of the 3-phase grounding current data of the iron core. The fault type is Phase A to ground. The blue, orange and green lines indicate Phase A, Phase B and Phase C’s grounding currents of the iron cores individually.

**Figure 3:**
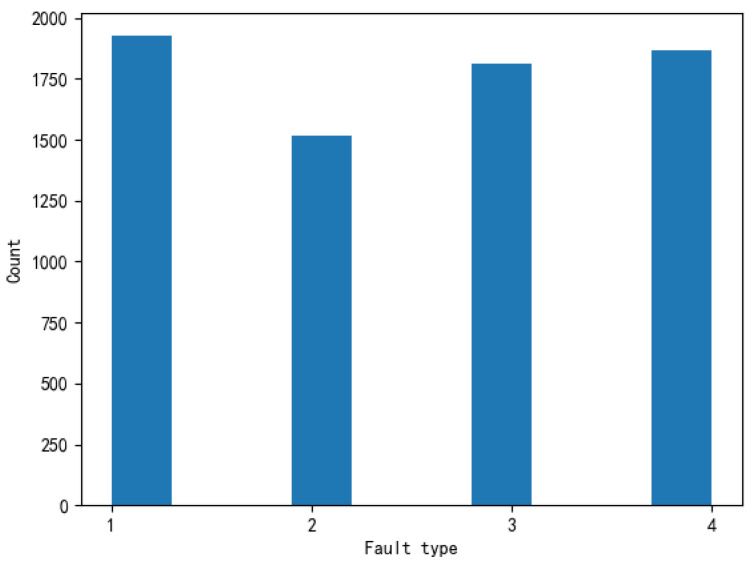
Distribution of the fault types in source domain.

The source domain collected four fault types’ data which are labelled. The next sample was one time-step shift from the last sample until the faults were fixed, where the time interval was twenty seconds. For example, the first sample was $D_{1}=\left\{X_{1,2,3...30}\right\}$ and the next sample was $D_{2}=\left\{X_{2,3,4...31}\right\}$. Figure 3 shows the distribution of all fault types in the source domain. The number 1 refers to Phase A to ground fault, 2 refers to Phase B to ground fault, 3 refers to Phase C to ground fault, and 4 refers to Phase ABC to ground fault. There were 1926 Type 1 fault data, 1516 Type 2 fault data, 1813 Type 3 fault data and 1867 Type 4 fault data.

#### 2.1.2. Target Domain

The target domain’s 3-phase fault grounding currents of the iron core data were collected from three 110 kV diverse power transformers. The detecting conditions, the time-steps and the data shape were the same as those of the source domain. The values of the fault data were similar to the source domain’s data.

The difference is that the fault types’ data of the target domain were combined with unlabelled and labelled types’ data. The labelled data were heavily unbalanced as well. Table 1 shows the distribution of fault type in the target domain of the three 110 kV different power transformers. Types 1, 2, 3 and 4 still refer to the same types as those of the source domain, and a Type 5 fault refers to unlabelled fault data. None means this kind of fault did not happen to this power transformer in the past year. The value means the sample size of each fault’s current data.

### 2.2. Preprocess

Data cleaning is the first step in data analysis. We pruned and checked the missing data and outliers due to the error of the detection device in the samples.

Three-phase currents are typical time series data. A time series *X* = $\left[x_{1},x_{2},\ldots ,x_{T}\right]$ is an ordered set of real values. The length of *X* equals the number of real values *T*. A key concept of time series prediction is that the data are expected to be stationary, which means the mean and variance of the data will not be varied too much with the passing of time. To improve the stationarity of the samples, we applied Lag2 differencing (1) to preprocess the data.

(1) d(t)=x(t)−x(t−n)

## 3. Model

**Figure 4:**
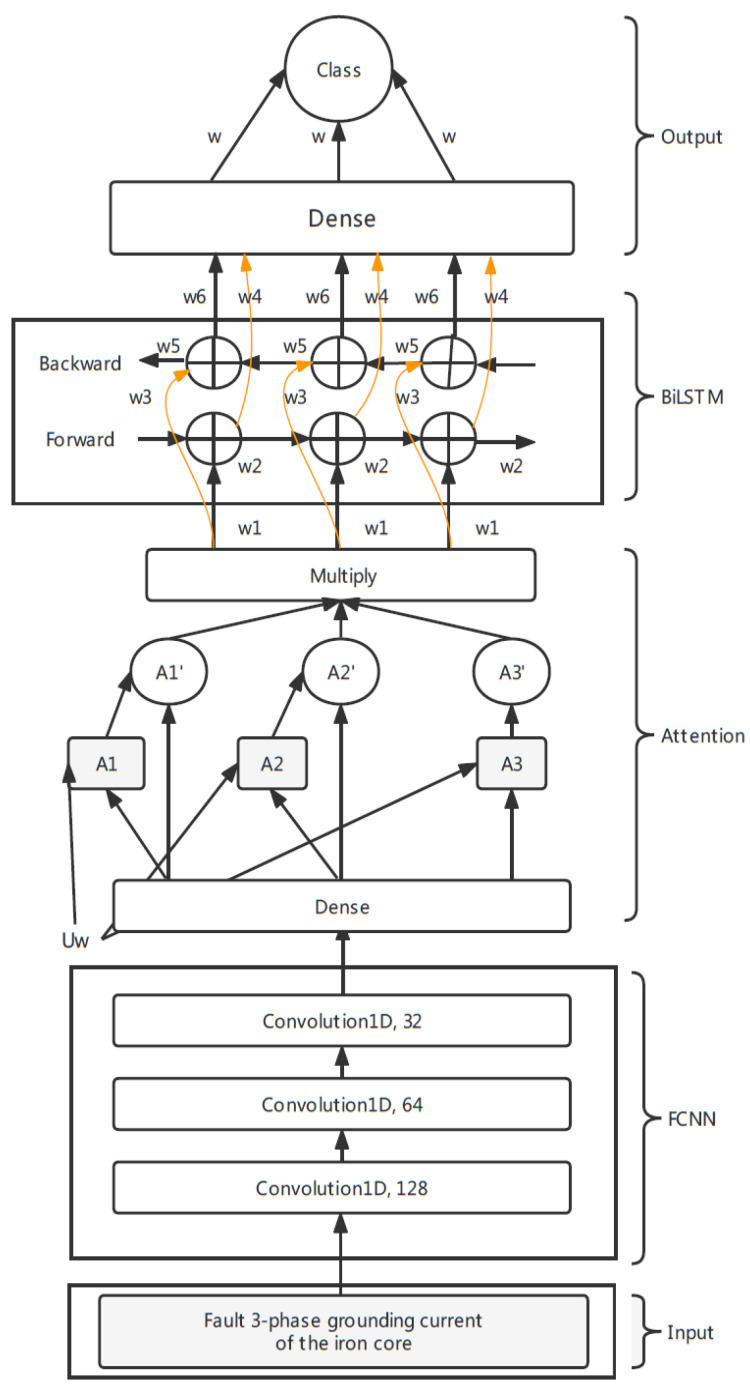
Model Architecture of FCNN–Attention–BiLSTM.

This section details the FCNN–Attention–BiLSTM model of transfer learning for fault classification in power transformers. The architecture of the model can be split into three parts: fully convolutional neural networks (FCNN), an attention mechanism and bidirectional long short-term memory (BiLSTM). The whole framework is shown in Figure 4. The input of the network is the three-phase grounding current of the iron core series data in the length of time_steps, which is 28 after Lag2 differencing. The network’s output is a probability distribution over the four possible fault classes.

### 3.1. Fully Convolutional Neural Networks (FCNN)

The first architecture is the one-dimensional fully convolutional neural network for its robustness, as it gains good performance in multiple series tasks to extract the hidden information. The input of the networks is the three-phase fault grounding current of the iron core data in the shape of time_steps×3. The first, second and third layers are convolutional layers with the rectified linear unit (Relu) [26] as an activation function for feature selection and each layer is followed by a batch normalization operation and dropout of 0.3. The first convolution is composed of 128 filters of length 1, the second convolution is composed of 64 filters of length 1 and the last convolutional layer is composed of 32 filters of length 1. The strides of the three convolutional layers are all equal to 1. The output of each convolutional layer is a multivariate time series in the shape of batch×time_steps×filter and will be normalized in the batch normalization layer and regularized in the dropout layer.

### 3.2. Attention Mechanism

The following is an attention mechanism [5]. The location of the attention is also important, as the different location of the attention captures different essential messages. The popular way is putting the attention layer after BiLSTM [7]. This paper adopts the method of transformed attention and sets the location of attention before BiLSTM but after FCNN to firstly extract feature importance.

(2) $$softmax\left(z_{i}\right)=\frac{e^{z_{i}}}{\sum_{c=1}^{C}e^{z_{c}}}$$

(3) $$\alpha_{i}=softmax\left(A_{i}U_{w}\right)$$

(4) $$c_{t}=\sum_{i}^{N}\alpha_{i}A_{i}$$

The first layer of attention is a dense layer with the activation functions of softmax (2). The dense layer’s input multiplies the dense output to complete the distribution of the weights. Because the input shape into the dense layer is (time_steps, input_dimension), where input_dimension is the number of units of the last layer of convolution, we then permute the shape into (input_dimension, time_steps), which is reasonable for computing the weights of each feature.

The feature at one time point is multi_dimensional, and the multiple features change with time, so the computed weight vector is multi_dimensional. This paper averages the weight vector to compute the attention score α after softmax is activated (3), where *A* is a matrix consisting of output vectors $\left[A_{1},A_{2},\ldots ,A_{N}\right]$ such that the FCNN layer is produced and *N* is the input_dimension.

The last step is repermuting the weight vector-matrix back into a (time_steps, input_dimension) shape and then multiplying the input (4). We add a dropout layer of 0.3 as well.

### 3.3. Bidirectional Long Short-Term Memory (BiLSTM)

The next layer is bidirectional long short-term memory (BiLSTM) [6], which is composed of two LSTM networks and can capture the information of the series from back to front. The LSTM neural network is formed by the input data $X_{t}$, cell state $C_{t}$, temporary cell state $\tilde{C_{t}}$, hidden state $h_{t}$, forget gate $f_{t}$, memory gate $i_{t}$ and output gate $o_{t}$ at time *T*. The computation process transfers the useful information from the cell state, drops the useless information and outputs the hidden state at each timestamp. The drop, memory and output are controlled by the last timestamp’s hidden state $h_{t-1}$, with the current forget gate, memory gate and output gate computed by the current input information $X_{t}$.

The joint of the forward LSTM outputs $\left\{h_{L0},h_{L1},...,h_{Ln-1}\right\}$ and backward LSTM outputs $\left\{h_{R0},h_{R1},...,h_{Rn-1}\right\}$ is the hidden state vector of BiLSTM. We set 512 neurons in this layer, which contains the information from front to back and from back to front. After that, we add a dropout layer of 0.3 to connect the final dense layer, whose activation is the softmax function (2).

The output of the model is a probability distribution of the four fault types generated by the dense layer. The fault is classified according to the highest probability.

## 4. Semi-Supervised Transfer Learning

**Figure 5:**
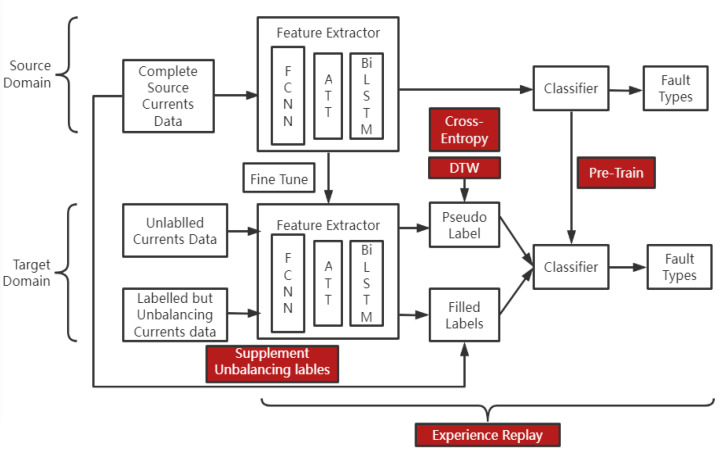
The framework of adaptive reinforcement (AR).

The whole framework of semi-supervised transfer learning, which we call adaptive reinforcement (AR), is shown in Figure 5 below. The source domain and target domain are detailed in Section 2. We split the architecture of the deep neural network into two parts: a feature extractor function $F_{\theta }$ and a classifier function $G_{\phi }$. $F_{\theta }$ is composed of the architecture as illustrated in Section 3, and we denote its weights in the pretrained model from the source domain as $\theta^{s}$. $G_{\phi }$ is the fully connected layer with respect to the fault types and we denote its weight in the pretrained model as $\phi^{s}$. The target dataset comprises labelled examples $D_{t}^{l}=\left\{x_{l}^{1,2...n}\right\}$, where *l* indicates labelled and unlabelled examples $D_{t}^{u}=\left\{x_{u}^{1,2...m}\right\}$, where *u* indicates unlabelled.

### 4.1. Pretrain

The first step of semi-supervised training is pretraining the target model. The feature extractor’s weights of the target model are initialized with $\theta^{s}$, the same as in the source domain’s model. We fine-tune their parameters in the later training process.

For the classifier’s weights of the target model, which we denote as $\phi^{t}$, we initialize them with random values for the weight columns and 0 for the bias column. Then, we fit the target model on the labelled examples $D_{t}^{l}$ with the Adam optimizer, whose learning rate is $1\times 10^{-7}$, and categorical-cross-entropy loss. We average the parameters of the last layer as the initial $\phi^{t}$. For example, the No. 1 power transformer has 1328 labelled data over 1840 samples. We fit the model whose weights are composed of $\theta^{s}$ and randomly initialize $\phi^{t0}$ with labelled samples (80% training dataset, 20% test dataset). Therefore, we obtain 1062 $\phi^{t1}$s and average the corresponding weight columns to obtain $\phi^{t1}$ and recompose it with $\theta^{s}$ as the target model.

### 4.2. Pseudolabel

Compared to other pseudolabelling methods [21,27], we design an algorithm to mark the labels based on cross-entropy and dynamic time warping (DTW) [28]. Cross-entropy is the distance between two probability distributions. If the distance is smaller, the two probability distributions are more similar. The DTW algorithm measures the distance between two series, and a smaller distance indicates higher similarity.

The source model’s output, composed of $F_{\theta }^{s}$ and $G_{\phi }^{s}$, is a probability distribution $P_{s}=G_{\phi }^{s}\left(F_{\theta }^{s}\left(x\right)\right)$, where x is the sample current’s data. $P_{s}$ is a one-dimensional vector with a length equal to the fault type number. For the the unlabelled sample data in the target domain, we compute each probability distribution $P_{s}^{u}$. Then, we calculate the cross-entropy (5) between $P_{s}^{u}$ with $P_{j}^{i,i\in 1,2,3,4}$.

(5) $$H\left(P_{s}^{u},P_{j}^{i}\right)=-\sum_{i=1}^{C}P_{s}^{u}logP_{j}^{i}$$

*C* is the classification number, which is 4 in our task. $P_{s}^{u}$ is the source model’s probability distribution of each unlabelled sample data. $P_{j}^{i}$ is the probability distribution of the fault type, for example, (1,0,0,0) for the Type 1 fault. We compute the cross-entropy values between each $P_{s}^{u}$ with the four $P_{j}$s. The lowest cross-entropy value over four results assigns the corresponding fault type to the unlabelled sample as the pseudolabel.

During the experiment, we found the cross-entropy values with different fault types were very close for a few unlabelled samples. For example, one unlabelled sample’s cross-entropy values with fault Type 2 and fault Type 3 are 0.34 and 0.35, separately. It would be unscientific to assign this sample to one type directly. We set an ε, which equals 0.2 if the difference between two cross-entropy values is less than ε (6), and we use DTW (7) to calculate the distance between the unlabelled samples with the confusing types of samples to determine the pseudolabel.

(6) $$\left|H\left(P_{s}^{u},P_{j}^{i}\right)-H\left(P_{s}^{u},P_{j}^{k}\right)\right|<\varepsilon$$

(7) $$DTW\left(Q,C\right)=min\left\{\frac{\sqrt{\sum_{K=1}^{K}w_{k}}}{K}\right\}$$

The principle of the DTW algorithm is that for two series *Q* and *C* with lengths *n* and *m*, we construct a matrix of n×m matrix where matrix (*i*,*j*) is the distance of $q_{i}$ and $c_{j}$, which is the Euclidean distance: ${\left(q_{i}-c_{j}\right)}^{2}$. We calculate the DTW distance between the unlabelled sample with all the confusing types of samples from the source domain and average the distances. The lowest distance, called the warping path $\left(w_{k}\right)$, assigns the unlabelled sample to the corresponding fault type.

### 4.3. Supplement Unbalancing Dataset

After pretraining and assigning the pseudolabels, we obtain a target model composed of $F_{\theta }^{s}$ and $G_{\phi }^{t}$ and a target dataset $D_{t}=D_{t}^{l}⋃D_{t}^{pl}$, the size of which was n+m. To make the distributions of the source and the target domain similar so that the model fit better, we supplement the target dataset with the weak types’ fault data from the source domain based on the probability distribution of the number of the fault types, as shown in (8).

(8) $$N_{s}^{w}=\frac{P(T=T_{s})}{P(T=T_{w)}}\times N_{t}^{w}$$

$N_{s}^{w}$ is the number of supplement samples to weak types’ samples, and $N_{t}^{w}$ is the number of weak types’ samples in the target dataset. $P(T=T_{s})$ is the probability of the most common fault happening in the target power transformer, and $P(T=T_{w})$ is the probability of the weak type’s sample in the target domain.

### 4.4. Experience Replay

The complete target dataset becomes $D_{t}=D_{t}^{l}⋃D_{t}^{pl}⋃D_{t}^{sup}$. $D_{t}^{w}$, the samples of the main fault type in each power transformer, are the most important. The mass occurrence of that type of fault over last year means this fault is more likely to happen. For a specific power transformer, we should consider the different conditions and adapt the weights more inclined to predict the main fault of that one. For this reason, we employ the idea of experience replay, which is used in the reinforcement learning model DQN [29].

For each batch during the training process, we denote the initial target model as *Q*, the sample of the most common fault as $x_{t}^{s}$ and the trained target model after the end of the training of each batch as $Q^{{}^{\prime }}$. We record the output vector in one dimension of the main fault’s sample as $P_{t}^{s1}=Q\left(x_{t}^{s}\right)$. Before moving on to the next batch, we replay the training process (9) as below.

(9) $$P_{t}^{s2}\left[s\right]=\gamma max\left(Q^{{}^{\prime }}\left(x_{t}^{s}\right)\right)+Q\left(x_{t}^{s}\right)\left[s\right]$$

We obtain a new output vector using the trained model *Q* for the specific sample during the replay process. We multiply the max value of the new output vector, which is the probability of predicting the most common fault, with a gamma which we set as 0.01. Then, we add the multiplication value to the raw vector’s corresponding fault probability as the new output vector $P_{t}^{s2}\left[s\right]$ for that sample and retrain the model $Q^{{}^{\prime }}$ on it.

## 5. Experimental Evaluation

This presents the results of the training processes on the source target and transfer learning results of the three target power transformers.

### 5.1. Model Training on the Source Domain

The source domain contains 7122 samples, which we shuffled randomly and split into 80% training and 20% validation sets. The model’s training process sets the batch sizes equal to 8 and 40 epochs with a loss function of categorical cross-entropy, Adam optimizer and accuracy metrics. The learning rate is changed during the training process since we found that the loss of train and validation datasets fluctuated after dozens of iterations. Therefore, we set the learning rate to be $1\times 10^{-3}$ for the first ten epochs, $1\times 10^{-4}$ for the following ten epochs, $5\times 10^{-5}$ for the next ten and $1\times 10^{-5}$ for the final ten epochs.

**Figure 6:**
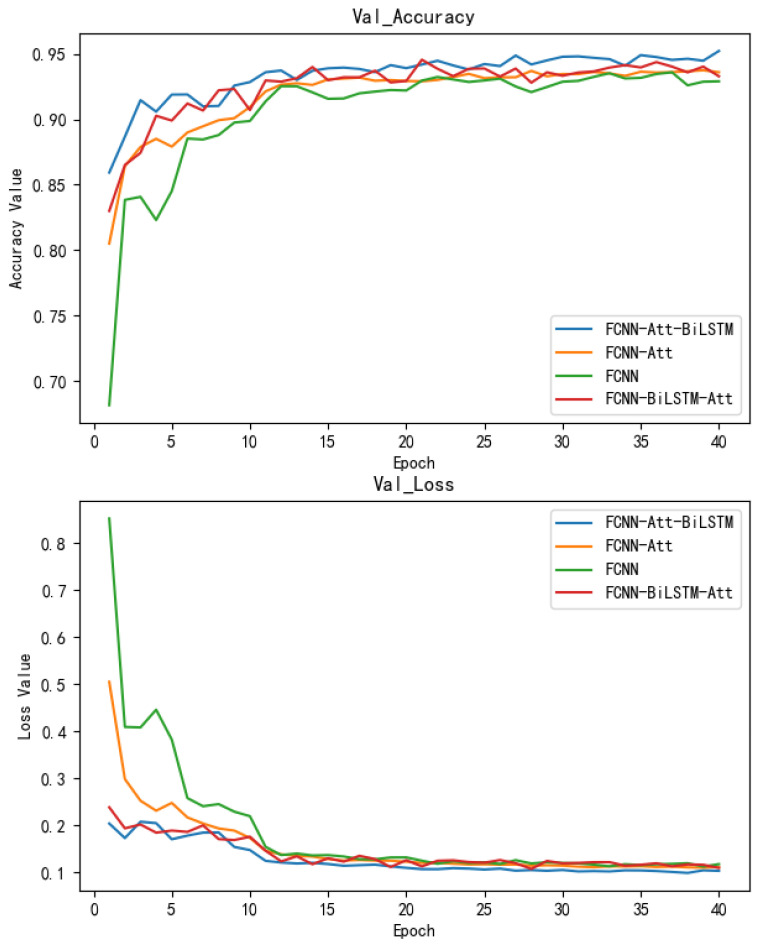
Training history of the validation set on the source domain.

The training history of the validation dataset is shown in Figure 6. We compare the proposed model architecture with three other models: FCNN–Attention, FCNN and FCNN–BiLSTM–Attention. The conditions of the training were all the same. We trained each model five times for fair conditions and picked the best one of them as the final model. The FCNN–Attention–BiLSTM mechanism outperformed the other three, ending with over 95% validation accuracy and about 0.1 validation loss.

For more Accuracy, we also calculated the Accuracy (10), Recall (11), Precision (12) and F1score (13) between the prediction values and true validation data. TP, TN, FP and FN stand for true positive, true negative, false positive and false negative, respectively. The evaluation results are shown in Table 2.

(10) $$Accuracy=\frac{TP+TN}{TP+TN+FP+TN}$$

(11) $$Recall=\frac{TP}{TP+FN}$$

(12) $$Precision=\frac{TP}{TP+FP}$$

(13) $$F1\;Score=2\times (\frac{Precision*Recall}{Precision+Recall})$$

### 5.2. Transfer Learning

We compare the proposed AR framework with the following state-of-the-art semi-supervised transfer learning methods. For a fair comparison, the learning process had the same framework of the learning rate as $1\times 10^{-5}$ because we did not want to suddenly change the weights of the model layers by a significant factor that may adversely affect the model performance, loss function, epochs and metrics.

1. Fine-tuning [18]: This is the pure supervised manner where unlabelled samples are deleted.
2. Pseudolabel [27]: It assigns the unlabelled samples by the model trained from the source domain.
3. Freeze [30]: This lets the feature extractor layers freeze and their parameters are untrainable during transfer learning. The unlabelled samples are ignored as well as fine-tuning.
4. ACR [21]: It contains the techniques of adaptive knowledge consistency by the entropy-gate algorithm and adaptive representation consistency by the maximum mean discrepancies (MMD) algorithm to regularize the weights.
5. EM [25]: The expectation maximization algorithm (EM) contains E and M steps. The posterior distribution is calculated in the E step by Bayes’s theorem. In the M step, the mixture distribution and the convolutional neural network parameters are updated.
6. LR [23]: It calculates the cross-entropy test loss and decomposes to several derivations, leading to a new regularization term called Lautum regularization (LR).

We also compare AR with and without the experience replay step as well. The aim of this research is trying to find the fastest and most steady transfer learning method. For this reason, we do not compare the final accuracy of each algorithm but the shortest time to achieve over 94% accuracy over three successive epochs on each target domain. The results of the acquired epochs for each transfer learning method is shown in Table 3. AR reached 94% accuracy with the least time consumption from retraining. In addition, the experience replay step accelerated the fitting process from the result.

The unlabelled data were given labels by the five semi-supervised transfer learning methods: pseudolabel, ACR, EM, LR and AR. We checked the target domain’s sample distribution after processing as shown in Table 4. Even after fitting the labels, the fault types were still unbalanced for the pseudolabel and ACR methods.

We predicted each raw target dataset without unlabelled samples to ensure that our model correctly predicted the raw dataset rather than overfitting. Then, we checked the accuracy, recall, precision and F1 score. The evaluation results are shown in Table 5. The accuracy is a little below the raw performance, but the difference is not big and can be affordable.

## 6. Discussion

The experiment was conducted on two tasks: the source task and the target task. The source task contained enough three-phase fault grounding currents of the iron core and balanced class type from one main 110 kV power transformer. The target task contained three 110 kV power transformers’ three-phase fault grounding currents of the iron core, while the target classes were unbalanced and contained undefined fault types. The three-phase grounding current of the iron core, Phase A, Phase B and Phase C, were recorded by three detection devices at the same time every twenty seconds. There were 7122 samples in the source domain and we preprocessed each one with the Lag2 difference method. We trained the proposed network on the source task in the framework of FCNN–Attention–BiLSTM and compared its performance with three other deep neural network frameworks: FCNN–Attention, FCNN–BiLSTM–Att and FCNN. For each model, we trained five times under the same conditions and recorded the best one. We visualized their training histories of them in Figure 6 and found out that our proposed model reached over 95% accuracy, which outperformed others. The model achieved 90% accuracy after four epochs but vibrates little. Its accuracy stabilized above 94% accuracy after 35 epochs and every epoch’s validation accuracy beat the other three models by more than 1–2% accuracy. For further comparison, we calculated the accuracy, recall, precision and F1 score of each model. The highest one was FCNN–Attention–BiLSTM, with about 95.37% accuracy, and the second one was the FCNN–Attention framework, with approximately 94.18% accuracy.

After we trained the model on the source task, we transferred it to the three target tasks. We compared our proposed semi-supervised transfer learning framework, AR, with six other state-of-the-art semi-supervised transfer learning methods: fine-tuning, pseudolabel, freeze, ACR, EM and LR. We also compared AR without the step of experience replay as well. The evaluation metrics were the fitting speeds of these frameworks. The quickest one was the AR framework, which just needed 9–10 epochs to stabilize over 94% accuracy at each target task, while the second one was the EM algorithm, which needed about 15 epochs. We also checked the new distribution of the fault type in each target task by the generating label methods: pseudolabel, ACR, EM, LR and AR. AR supplemented all the lacking type’s data so it required more time to train each epoch, though the time consumption of fitting was still the lowest. When the model stabilized over 94% accuracy, we stopped training and predicted the validation set’s classes with the raw dataset without the supplement samples. The accuracy for the three power transformers was about 93% separately. Though it is a little lower than the accuracy on the training dataset, the difference is less than 1%, which is affordable.

The aim of this investigation is classifying fault type after fault occurrence based on the three-phase grounding current of the iron core. The work focuses on quick semi-supervised transfer learning. For further research, we will try to predict fault preoccurrence with the dissolved gas data. The methodology can be improved with signal processing techniques, the frequency response analysis technique and loss optimization algorithms.

## 7. Conclusions

This research focuses on the task of classifying the fault types, Phases A, B, C and ABC to ground fault, based on the three-phase fault grounding current of the iron cores from 110 kV power transformers. The values of the data were recorded every twenty seconds and increased substantially after the fault occurred. After the abnormal current appeared and exceeded the warning threshold, which was 100 mA, we collected the sample of the fault current in the length of 30 and preprocessed it with the Lag2 difference method.

This paper proposes an FCNN–Attention–BiLSTM model to classify fault type based on fault data. This architecture achieves over 95% accuracy. Apart from this, a semi-supervised transfer learning method called AR is illustrated to transfer the trained model to diverse power transformers. AR includes four steps: pretraining the classifier layer, generating a pseudolabel by DTW and cross-entropy algorithms, supplementing unbalanced data by the probability distribution of the fault classes and relaying the training experience of the most-occurring fault. AR reduces the epochs required dramatically to achieve a high accuracy score in our target task.

For real-time diagnosis, while the current value exceeds the warning threshold, which is 100 mA, the system waits until enough data in the length of 30 (10 min from when the fault happens) are generated. The model inputs the preprocessed fault data and returns the probability distribution of the four types of faults. If the largest probability is higher than the confidence level, which we set as 0.7, the system will deliver the corresponding fault type message to the maintenance worker. On the other hand, if the four probabilities are close, the system will wait another twenty seconds until the next detection data are generated and then replicate the above steps.

## Figures and Tables

**Table 1:** Fault distribution of the target domain.

Power Transformer	Type 1	Type 2	Type 3	Type 4	Type 5
No. 1	164	None	1107	57	512
No. 2	None	176	113	1319	413
No. 3	None	872	None	491	634

**Table 2:** Evaluation results of different deep learning models’ performances on the source domain.

Evaluation/Methods	FCNN–Att–BiLSTM	FCNN–Att	FCNN	FCNN–BiLSTM–Att
Accuracy	95.37%	94.18%	93.33%	93.63%
Recall	95.38%	94.19%	93.33%	93.63%
Precision	95.37%	94.18%	93.33%	93.63%
F1 Score	95.37%	94.18%	93.33%	93.63%

**Table 3:** Epochs required to achieve over 94% accuracy three times for each transfer learning method.

Methods/Transformer	No. 1 Power Transformer	No. 2 Power Transformer	No. 3 Power Transformer
Fine-tuning	24	25	29
Pseudolabel	26	27	32
Freeze	35	37	35
ACR	13	15	15
EM	13	11	13
LR	14	15	14
AR without experience replay	14	13	14
AR	9	9	10

**Table 4:** Sample distribution of each power transformer.

	No. 1 Power Transformer				No. 2 Power Transformer				No. 3 Power Transformer			
Methods/Labels	Type 1	Type 2	Type 3	Type 4	Type 1	Type 2	Type 3	Type 4	Type 1	Type 2	Type 3	Type 4
Pseudolabel	287	113	1107	333	189	259	175	1398	347	913	122	615
ACR	357	97	1188	198	341	212	143	1325	280	954	165	598
EM	291	143	1217	189	303	271	183	1264	263	931	186	617
LR	350	103	1174	213	339	225	140	1317	291	944	169	593
AR	1347	1347	1347	1347	1366	1366	1366	1366	993	993	993	993

**Table 5:** Evaluation results of our proposed semi-supervised transfer learning models after training on the raw target datasets without unlabelled samples.

Evaluation/Methods	No. 1 Power Transformer	No. 2 Power Transformer	No. 3 Power Transformer
Accuracy	93.13%	93.27%	93.07%
Recall	93.13%	93.27%	93.07%
Precision	93.14%	93.27%	93.07%
F1 Score	93.13%	93.27%	93.07%

## Data Availability

Not applicable.
